# Building-blocks to develop one health systems

**DOI:** 10.1016/j.onehlt.2023.100624

**Published:** 2023-08-25

**Authors:** Paulo Ferrinho, Cláudio Tadeu Daniel-Ribeiro, Rosa Ferrinho, Inês Fronteira

**Affiliations:** a Global Health and Tropical Medicine, Instituto de Higiene e Medicina Tropical, Universidade Nova de Lisboa, Rua da Junqueira, 100, 1349-008 Lisbon, Portugal; b Laboratório de Pesquisa em Malária, Instituto Oswaldo Cruz & Centro de Pesquisa, Diagnóstico e Treinamento em Malária, Fiocruz, Ministério da Saúde, Brazil; c Escola Superior de Saúde, Bem Estar e Proteção Animal, Instituto Politécnico para a Lusofonia, R. do Telhal aos Olivais 8, 1950-396 Lisbon, Portugal; d NOVA National School of Public Health, Public Health Research Center, Comprehensive Health Research Center, CHRC, NOVA University Lisbon, Escola Nacional de Saúde Pública, Avenida Padre Cruz, Lisbon 1600-560, Portugal; e Laboratório de Pesquisa em Malária, Instituto Osvaldo Cruz, Fiocruz, Pavilhão Leónidas Deane, 5° andar, Av Brasil 4365, Rio de Janeiro CEP 21.041-250, RJ, Brazil

**Keywords:** One health, One health systems, Building-blocks

## Abstract

Notwithstanding the understandable rationale of the logical, expected and natural evolution of human behaviour towards an anthropocentric view of its relationship with other animals and the environment, a shift from this predatory “Ego-centric” behaviour towards an “Eco” conduct, with regard to their view of the world and of the global health, has become mandatory, contributing to the development of the “One Health” and of “One Health Systems” concepts. We contend for the usefulness of a building-blocks approach to facilitate an understanding of the development of One Health Systems. We assert that a building-blocks approach to One Health Systems with strong similarity to WHO's building-blocks for human health systems would help to strengthen the case for robust,resilient and anti-fragile One Health systems.

## 1 Introduction

It is reasonable to consider that the history of humanity has been marked by two transformational revolutions: the cognitive revolution (35,000 to 70,000 years ago), when *Homo sapiens* began to manifest our capacity to abstract, imagine things that did not exist and give meaning to non-representational thoughts; the agricultural revolution (10,000 to 12,000 years ago), when *Homo sapiens* stopped being a hunter/collector to settle on land, cultivating the soil and raising domesticated animals. It was possibly the beginning of property ownership, monogamy, family and also when malaria, and other diseases more easily spread in communities, became consolidated as human diseases [1].

Until the cognitive revolution we could be classified as “insignificant animals… that did not change our planet any more than gorillas … and fireflies” [1]. It was with the cognitive revolution that things changed and our previously acquired abilities of creating images and dealing with abstract concepts started to push humankindto become the “Dominant Mammal” controlling all other animals and Earth's environments. This occurred probably as a result of several factors including bipedalism and consequent freeing of the hands, and development of precision and mastering of fine movements with the opposable thumbs, privileged intelligence and, mainly, of modern cognition *modus operandi* [2].

Progressively, man learned to work with wood, iron and all minerals extracted from the soil, produced clothes with leather and wool from animals, linen and cotton from plants, silk from insects, and latter from natural oils created arts, jewelry, dynamite, electricity, vaccines, transatlantic ships, locomotives, cars, computers, antibiotics, contraceptives, robots, portable cell phones, Viagra, informatics, bioengineering, internet, artificial intelligence, social networks and many other innovations [3].

Not surprisingly, conscious of all these attributes, man felt important, central and essential, falling into the temptation of judging himself a magnificent creature with all rights and that became deep rooted in some cultures advocating for a “earth centrism” as was the case of the Judeo-Christian thought for many centuriesIn his search for pleasure, comfort, sophistication and power, man interfered with and altered the environment in a so ambitious and predatory manner that he modified and compromised his and other species living conditions, causing also climate changes that threaten his own survival on the planet with “not a square meter of land in the temperate regions whose appearance has not been shaped by man” [4].

With time, it became clear that, in addition to this entirely anthropocentric “Ego's” point of view, there might be another perspective from which one can perceive the world and interact with it. An ecological “Eco's” perspective corresponds to a compassionate view (how can we contribute to the greater good and make the world a better place) of the Globe. After all the damage done to nature by man, moving from the so-called Ego to the Eco attitude became a mandatory need [5].

Concerning Global Health, for a long-time, human health has benefited by sacrificing the “health” of wild ecosystems (e.g., deforestation and conversion of wilderness to farmland, damming of water for irrigation, destruction of swamps and dislocation and jeopardy of wild species) which reflects a protectionist vs utilitarian conflict, over the question of whether to put human domination of the biosphere on hold or whether to embrace it [6]. It hides unaddressed concerns about the value of (macroscopic) life (human, animal or plant), the definitions of health and wellbeing (human, animal, plant and environmental) and their relative importance [7,8]. This has hampered the development and acceptance of a One Health (OH) understanding of Global Health [9].

## 2 Definitions of one health

The representation of OH is as an umbrella [10]. It retains definitions of health around which the sectorised health systems have developed: human health, animal health, wildlife health, plant health and environmental health, overlooking that health, in an ecological context, encompasses the health of all organisms, including humans, interconnected within ecosystems in which they live [11].

We argue the need to move beyond the umbrella metaphor, towards a more systemic and syndemic understanding of OH which would allow to eventually share an understanding of “One Health Systems” (OHS) in the same way that, in human health, we refer to health systems. Following a proposal made by European Ministers for Foreign Affairs at the 2020 Paris Peace Forum, in May 2021, the interdisciplinary One Health High-Level Expert Panel (OHHLEP) was created with “the intent to take this concept forward into policies and concrete actions” [7].

Nevertheless, the lack of a precise definition made OH impractical as a policy target for countries. Therefore, an immediate priority for the Panel was to develop consensus around a working definition of OH. The OHHLEP definition, adopted in this paper, mobilizes multiple sectors, disciplines, and communities at varying levels of society to work together to foster well-being and tackle threats to health and ecosystems, while addressing the collective need for healthy food, water, energy, and air, taking action on climate change and contributing to sustainable development” [7].

## 3 Advent of the concept one health systems

OHS have been developing gradually and elusively, not following the unravelling of a classical health system, but rather the notion of a fuzzy logic of systems for an ill-defined concept of health [12].The advent of the term OHS in the literature, dating only from 2017, is even more recent than the term OH [[13], [14], [15], [16], [17], [18]].

OHS should build on the development of processes that can be used to “foster synergies across agencies and improve multi-sectoral preparedness, detection, and response to complex One Health challenges” [19]. These processes “are often siloed and frequently sectoral”, leading to calls on governments “to promote the implementation of roadmaps” with the priority “to engage sectors in a systems-based approach at national/subnational levels” to strengthen OHS in countries [18,[20], [21], [22]]. We propose an understanding of OHS constructed on a building-blocks approach.

Building-blocks offer modular components that may be relied on to work together and grow coherently as the pieces making up a system. Blocks help to focus design efforts on the important questions of what content to address, how to present it to policy makers, and how to manage it effectively. They facilitate the development of services across organizations and borders. A building block integrating a system must interoperate with other building-blocks [23,24].

The building-blocks concept has been adapted by the WHO to human health systems, conceptualizing these based on six building-blocks: service delivery, health workforce, health information systems, access to essential medicines, financing, and leadership/governance (Table 1) [24]. Since its launch in 2007, this framework has been widely considered instrumental in strengthening human health systems, and as a catalyst for achieving global health targets such as the Sustainable Development Goals [25,26].

**Table 1:** WHO's health systems' building blocks.

Building block	Scope
Service delivery	Is an immediate output of the inputs into the health system, such as the health workforce, procurement and supplies, and financing. Increased inputs, if framed by appropriate policies and strategies (governance), should lead to improved health service delivery and enhanced access to services. Ensuring availability of health services that meet a minimum quality standard and securing access to them is a key function of a health system.
Workforce	Attaining health goals depends largely on the knowledge, skills, motivation and deployment of the people responsible for organizing and delivering services. Many countries, however, lack the human resources needed to deliver integrated health interventions for a number of reasons, including inadequate planning, limited relevant production capacity, poor mix of skills and demographic imbalances. The formulation of health national policies and plans in pursuit of human resources for health development objectives requires sound information and evidence. Against this backdrop of an increasing demand for information, building knowledge and databases on the health workforce requires coordination across human, animal and environmental sectors. Various permutations and combinations of what constitutes the health workforce may exist according to the country's situation and the means of monitoring.
Information and research	In addition to being essential for monitoring and evaluation, the information system also serves broader objectives, such as providing an alert and early warning capability, supporting health facility management, enabling planning, underpinning and stimulating research, permitting health situation and trends analyses, orienting global reporting, and reinforcing communication of health challenges to diverse users.
Financing	Refers to the function of a health system concerned with the mobilization, accumulation and allocation of money to cover health needs, in order to make funding available, as well as to set the right financial incentives for health system development.
Products and technologies	Refer to equitable access to essential pharmaceutical products, vaccines and technologies of assured quality, safety, efficacy and cost-effectiveness, and their scientifically sound and cost-effective use.
Leadership/ governance	Involve ensuring that policy frameworks exist and are combined with effective oversight, coalition-building, regulation, attention to system design and accountability. Accountability is an intrinsic aspect of governance that concerns the management of relationships between various stakeholders, including individuals, households, communities, professional organizations, firms, governments, nongovernmental organizations, private firms and other entities that have the responsibility to finance, monitor, deliver or use health services.

Source: Adapted from World Health Organization, 2016 [27].

## 4 Building-blocks and one health systems

A World Bank Report has advocated “foundational building-blocks to develop OH interventions that may be implemented at varied levels of specificity (…) or broadness”. These building-blocks are presented separately in distinct stages (prevention, detection, response and recovery) and include stakeholders, roles, and responsibility; financial and personnel resources; communication and information; technical infrastructure; and governance (Table 2) [17]. Their overlap with some of the building-blocks proposed by WHO is striking.

**Table 2:** World Bank's One Health Systems' building blocks.

Building block		Scope
Governance		Prevent through legally required reporting to national authorities in order to inform risk analysis so that there are no gaps in the relevant authority, so that disease risk is included in the environmental and social impact assessment and risk mitigation is incorporated into high-risk practices, as well as economic evaluation of risk management options. Initial communication to national and international authorities in order to respond to the outbreak, by updating it and adapting its risk level. Recover through demonstration of indemnity status and biosecurity regulation.
Stakeholders, roles, and responsibilities		Prevention through technical entities that conduct research, sectoral and geographic distribution of active surveillance, risk assessment, planning and implementation of health disaster risk reduction through mitigation and surveillance resources. Detection through technical and non-technical entities contributing to passive surveillance, including private sector networks, distribution of laboratory services and reporting channels and resources for laboratory services. Response using technical and non-technical entities within public health and healthcare systems, e.g. hospitals, government epidemic investigation teams, civil society, non-governmental organizations and other groups, including the private sector, affected by a disease, e.g. as well as well as entities for emergency funding and resources for outbreak investigation/control and treatment. Recovery through changes in mandates and chain of command, also taking into account the role of the private sector in resilience and providing resources for recovery.
Resources	Financing	Routine and contingent funds that allow for an enhanced allocation of resources based on the deficits identified in baseline assessments, thus enabling emergency mobilization for treatment, research, containment and control, as well as a check on the emergency budgetary capacity available and mobilized. Origin of these funds are government budgets, research grants and development projects. The aim of these funds is to increase the allocation of resources based on the shortfalls identified.
	Personnel	Specialized technicians in different fields of sciences and research, prioritizing the human, animal and environmental health and social sciences
Communication and information		Access to information for risk assessment and mitigation, drawing on lists of pathogens in the country, lists of known disease hosts and reservoirs in the country, pre-identification of country exposure and regional risk profile, including through meteorological data on climate-sensitive diseases, established contacts between ministries, chain of command for reporting and action, specific and population-sensitive messages. Pre-identification of risk factors likely to facilitate spread and multi-sectoral awareness raising. Continuous coordination between authorities and between relevant ministries, affected sectors, logistical actors, media and civil society by multi-sectoral resilience planning and priority setting and after-action analysis and improved communication/information.
Technical infrastructure		Prevent through national, regional, or international access to laboratory diagnostics, sentinel surveillance on animals or vectors and hazard investigation and identification and other relevant stages of risk analysis, as well as risk mitigation and identification of vulnerable populations. Detect through national access to laboratory diagnostics, reference laboratory confirmation analysis if needed, disease prioritization, detection at point of entry. Strengthen health systems, including through medical treatment, control at point of entry, and preventive measures to reduce the potential for cross-border spread. Risk mitigation measures, e.g. universal vaccination campaigns, climate-resilient and other health care infrastructure, improved risk assessment, provision of continuous medical treatment, and biosafety facilities and personnel.

Source: World Bank and EcoHealth Alliance [17].

The ‘One Health Joint Plan of Action (2022–2026)’ acknowledges “professional segregation with limited cross-sectoral working, inadequate representation of some sectors, disjointed legislative schemes, a lack of data sharing and transparency, an absence of multisectoral coordination mechanisms, siloed budgets and decision-making processes, and a lack of robust regulatory frameworks, legal support, mandates and enabling policies are additional barriers hindering the effective implementation of OH (…) OH requires (…) effective governance rooted in transdisciplinary and multisectoral principles and appropriate legislation, stakeholder and community engagement, and the integration of the concept into education in related disciplines”. Although less assertive than WHO's or the World Bank's building-blocks, it is still possible to glimpse five building-blocks behind the theory of change adopted in the plan (Table 3) [18].

**Table 3:** One Health Joint Plan of Action (2022–2026) building blocks.

Building block	Scope
Governance	Policy development, political will, enabling regulatory frameworks, investment and the institutionalization of intersectoral governance (pathway 1 and Part 4 of the action plan)
Stakeholder and community engagement	Several sections of the action plan and pathways emphasize the importance of stakeholders. As examples, we emphasize the need to promote awareness, policy changes and action coordination among stakeholders to ensure that humans, animals and ecosystems achieve health and remain healthy in their interactions with and along the food supply chain. This requires continued institutionalization, mobilization and better use of resources across sectors and stakeholders, supported by appropriate investments for greater awareness among all stakeholders. Overcoming lack of cooperation between internal and external stakeholders, limited engagement with the environmental sector and professional segregation is a major priority. Empowering stakeholders, including civil society, disadvantaged groups and indigenous communities and local stakeholders acknowledges their central role in the identification of the local challenges and in the design and implementation of locally adapted OH solutions, with the support of guidelines for stakeholders to design joint processes for OH. Communication remains a central strategy that should take into consideration the different goals and benefits that stakeholders share.
Implementation	Organizational development, implementation and sectoral integration – encompasses all aspects of the implementation of OH, including the scaling up of capacity development at regional and country level, community engagement and mobilization for action, multisectoral coordination, collaboration and communication, and the equitable integration of sectors (pathway 2 and Part 4 of the action plan)
Monitoring and evaluation	Data, evidence and knowledge – encompasses the strengthening of the scientific evidence base, knowledge translation into data for evidence, technical tools, protocols and guidelines, information and surveillance system (pathway 3 and Part 4 of the action plan)
Resource mobilization	Mobilize and make better use of resources across sectors, disciplines and stakeholders (Part 5 of the action plan).
	Education in related disciplines

Source: Adapted from FAO et al. [18].

## 5 Conclusions

Notwithstanding the understandable rationale of the logical, expected and natural evolution of human behaviour towards an anthropocentric view of its relationship with other animals and the environment, a shift from this predatory “Ego-centric” behaviour towards an “Eco” conduct, with regard to their view of the world and of the global health, has become mandatory.

It is, however, consensual that considering animal, human and environmental health separately and not the health of ecosystems in its most seminal understanding, within narrow perspectives is no longer valid. At an international level, agreement on OH principles and tools have progressed to the point of joint action plans, such as ‘One Health Joint Plan of Action (2022–2026)’ already referred to. At a national level in almost all countries, ministries remain separate and sectoralised, with their own budgets and agendas without integration of health programmes: countries usually have animal health programs, human health programs but rarely OH programs.

We contend for the usefulness of a building-blocks approach to facilitate an understanding of One Health Systems' development. If we compare the building-blocks addressed in this viewpoint, we dare to assert that a building-blocks approach to OHS with stronger similarity to WHO's building-blocks for human health systems would help to strengthen the case for robust, resilient and anti-fragile OHS. “Workforce”, “health products and technologies” and “service delivery” are addressed in the OHS literature, but not with the differentiation and assertiveness that their importance requires (Table 4).

**Table 4:** Comparative approaches to Human/One Health system's building-blocks.

Institution	WHO	World Bank	One Health Joint Plan of Action (2022–2026)
Focus	Human health	One health	One health
Building-blocks	Leadership/ governance	Governance	Governance
	Not acknowledged as a building block	Stakeholders, roles, and responsibility	Stakeholders and community engagement
	Financing	Resource mobilization (financial personnel)	Resource mobilization/education
	Workforce		
	Products and technologies		
	Information and research	Information and research	Monitoring and evaluation
	Service delivery	Technical infrastructure	Implementation

Source: The authors.

Since its launch in 2007, some authors argue that WHO's framework should be improved by integrating the missing “demand” component; incorporating an overarching, holistic health systems viewpoint; including explicit considerations to decision-making and power; considering scope for interactions between components; adding a meaningful inter-sectoral collaboration block; and putting in a functioning global health surveillance and response system [22,29].

These, and probably other, amendments, such as stakeholders and community engagement, would contribute to increase the areas of overlap among the three sub-systems, moving to a really shared systems approach that would contribute to further the OHS cause, where the differentiating elements of the different sub-systems (human, animal, plant and environmental) would be ontological and methodological, rather than teleological or deontological.

## Declaration of Competing Interest

The authors declare that they have no known competing financial interests or personal relationships that could have appeared to influence the work reported in this paper.

## Data Availability

No data was used for the research described in the article.
